# Characterization of Exopolysaccharides from *Lactiplantibacillus plantarum* PC715 and Their Antibiofilm Activity Against *Hafnia alvei*

**DOI:** 10.3390/microorganisms12112229

**Published:** 2024-11-03

**Authors:** Xiqian Tan, Bingyu Ma, Xiaoqing Wang, Fangchao Cui, Xuepeng Li, Jianrong Li

**Affiliations:** 1 College of Food Science and Engineering, Tianjin University of Science and Technology, Tianjin 300457, China; 2 College of Food Science and Engineering & Institute of Marine Science and Technology, Bohai University, Jinzhou 121013, China

**Keywords:** *Lactiplantibacillus plantarum*, exopolysaccharide, structural characterization, antioxidant activity, antibiofilm, *Hafnia alvei*

## Abstract

Exopolysaccharides (EPSs) secreted by lactic acid bacteria have the potential to enhance human health by showing various biological functions. This study investigated the biological role and antibiofilm properties of EPS715, a new neutral EPS produced by pickled vegetables originating from *Lactobacillus plantarum* PC715. The results indicate that EPS715 is primarily composed of rhamnose, glucose, and mannose. Its molecular weight (Mw) is 47.87 kDa, containing an α-glucoside linkage and an α-pyranose ring. It showed an amorphous morphology without a triple helix structure. Furthermore, EPS715 showed improved antioxidant activity. Specifically, its scavenging capacity of ABTS^+^ radicals, DPPH radicals, and the hydroxyl (·OH) reduction capacity at 5 mg/mL was 98.64 ± 2.70%, 97.37 ± 0.79%, and 1.64 ± 0.05%, respectively. Its maximal scavenging capacity was >40%, and the hydroxyl (·OH) radical scavenging ability was dose-dependent. Moreover, the biofilm of various pathogens including *S. aureus*, *B. cereus*, *S. saprophyticus*, *Acinetobacter* spp., and *H. alvei* was substantially dispersed and affected by EPS715, with a maximum inhibition rate of 78.17% for *H. alvei*. The possible mechanism by which EPS715 shows antibiofilm properties against the *H. alvei* may be attributed to its effects on the auto-aggregation, hydrophilic characteristics, and motility of *Hafnia* spp. Thus, EPS715 has significant antioxidant and antibiofilm characteristics that may hold substantial potential for applications in food and medicinal products.

## 1. Introduction

Microbial exopolysaccharides (EPSs) are large molecular polymers secreted from the cell during specific microbial growth. These polysaccharides can be categorized into two types: homogeneous polysaccharides (Hops) and heterogeneous polysaccharides (Heps) [1]. Hops consist of a single type of monosaccharide, whereas Heps comprise many monosaccharide components. Microbial polysaccharides are predominantly classified as Heps. Studies have found that polysaccharides derived from different microbes demonstrate distinctive structures, resulting in diverse functions. Commonly, EPSs can be used as a thickener or emulsifier in the industry, improving the taste and flavor of food [2]. They also serve various biological functions along with their industrial applications. For example, the EPS of *Bacillus licheniformis* AG-06, which is derived from the polyherbal fermented medicinal product *Ashwagandharishta* in Indian Ayurveda, comprises galactose, xylose, mannose, rhamnose, and glucose. It possesses a fibrous and porous web-like structure and demonstrates potent antioxidant and antiproliferative properties [3]. Furthermore, EPS-2, a biopesticide that is primarily composed of mannose, glucose, rhamnose, D-GalN (8.0%), GlcN (35.5%), Man-UA (0.3%), arabinose (5.1%), and Glc-UA (0.2%), is derived from *Bacillus thuringensis* 4D19. Its distinctive morphology is characterized by rice-shaped grains that further aggregate into a network structure on the surface [4].

Lactic acid bacteria, particularly *Lactiplantibacillus plantarum*, are another major probiotic that can potentially secrete EPS. Furthermore, the composition, structures, and functions of monosaccharides vary among different strains. Most EPSs secreted by *L. plantarum* consist of galactose and glucose [5] as well as mannose, rhamnose, and arabinose [6]. Some EPSs contain xylose such as that produced by *L. plantarum* WLPL04 [7]. Most *L. plantarum* EPSs have revealed antioxidant ability. Some *L. plantarum* EPS derivatives, like acetylated EPS, carboxymethylated EPS, and sulfated EPS, have stronger antioxidant activities. Many *L. plantarum* EPSs also possess unique characteristics such as anti-inflammatory [8], enhancing intestinal barrier function [9] antibacterial and antibiofilm [10] activities, etc.

Biofilms are a community of microorganisms that accumulate on the surface, thereby compromising food safety and negatively impacting food processing equipment. Moreover, compared to planktonic cells, biofilms are resistant to antimicrobial agents and are, therefore, more difficult to remove [11]. To date, except for *L. plantarum* EPS, studies have shown that EPSs from other microorganisms also have antibiofilm features against different pathogens. Current research suggests that the antibacterial role of EPSs may be demonstrated through various mechanisms including altering the physicochemical properties of surfaces in contact with bacteria, competing with bacterial carbohydrate–protein interactions, having bactericidal effects, and influencing the expression of critical genes involved in bacterial biofilm regulation. Initially, many EPSs can enhance the hydrophobic properties of abiotic surfaces, thereby inhibiting cell aggregation. For example, the marine bacterium *Vibrio* MO245 secretes EPSs that resemble hyaluronic acid. Based on the cyclical characteristics associated with biofilm formation, MO245 has the potential to form a non-adsorptive coating on surfaces, which can impede the early adhesion of bacteria [12]. Meanwhile, the EPSs from human-origin *L. plantarum* EIR/IF-1 act as a surfactant, preventing the adhesion of negatively charged Gram-negative bacterial cell membranes at the junction via its negatively charged surface [13]. However, EPSs can interfere with the interactions between carbohydrates and proteins within bacteria. Some EPSs can act as lectin (a type of agglutinin) inhibitors. Lectins are proteins that recognize and bind sugars without altering the molecules. In bacteria, the primary role of lectins is to facilitate adhesion. EPSs can competitively inhibit the sugar-binding domains on lectins, thereby obstructing the normal adhesion process of the cells [14]. Furthermore, several studies have identified various EPSs with antibacterial properties. For instance, the EPS produced by *Lacticaseibacillus paracasei* AS20 can inhibit *Listeria monocytogenes*, *Yersinia enterocolitica*, and *Bacillus cereus* [15]; EPS from *L. plajomi* PW-7 can inhibit *Helicobacter pylori* [16]. Most of these antibacterial EPS are Heps, and their antimicrobial effects (i.e., MIC and MBC values) and their ability to inhibit biofilm formation are related to the structure of the polysaccharides and the type of target strains.

*Hafnia alvei* is a highly representative type of spoilage organism (SSO) found in seafood, and its presence can lead to the spoilage of vacuum-packaged aquatic products. It has a strong adaptability to low temperatures [17,18], which poses a challenge since vacuum packaging and low-temperature storage and transportation are currently common and cost-effective methods for preserving seafood. Therefore, it is necessary to investigate control strategies targeting *H. alvei* [19]. Biofilm formation is a robust capability of many SSOs such as *H. alvei.* The formation of biofilms is associated with the production of virulence factors and the emergence of degradation capacities, which increases their resistance to antimicrobial agents and unfavorable environments. Therefore, it becomes more difficult to remove them from the surfaces of shrimp shells and fish scales, packaging products, and seafood processing equipment [20]. Thus, this raises the safety risks related to consumption and requires a risk of seafood spoilage. To date, no research is currently available to investigate the antibiofilm properties of the *L. plantarum* EPSs against the biofilm of *H. alvei*.

This study investigated the EPS isolated from a pickled vegetable originated *L. plantarum* PC715, characterized its structure, and explored the antioxidant and antibiofilm abilities, especially the potential antibiofilm mechanism of EPS715 against *H. alvei*. Therefore, this study establishes a theoretical basis for applying EPS715 as a biofilm inhibitor in fishery products.

## 2. Materials and Methods

### 2.1. Reagents

Trichloroacetic acid (TCA, 99.0%; CAS:76-03-9) was procured from Aladdin Biological Technology Co. Ltd. (Shanghai, China). Standard sugars were procured from Sigma-Aldrich (Shanghai, China). Antifade mounting medium (with DAPI) was purchased from Coolaber Technology Co. Ltd. (Beijing, China). The remaining reagents/chemicals used in the current study were analytical grade. *L. plantarum* PC715 was isolated from naturally fermented pickled vegetables, the growth of the strain on the DeMan, Rogosa, and Sharpe (MRS) broth supplemented with 5% sucrose is shown in Supplementary Figure S1, and the EPS was secreted around the strain. *L. plantarum* PC715 was preserved at the China General Microbiological Culture Collection Center (accession number CGMCC No: 28266).

### 2.2. EPS Purification

*L. plantarum* PC715 was cultured in MRS broth medium (2%, *v*/*v*) supplemented with 50 g/L sucrose at 37 °C for 48 h, with agitation at 160 rpm. After culturing, the culture was spun at 10,000 r/min for 20 min at 4 °C to collect the supernatant. Next, the EPS was precipitated by adding 3-fold volumes of 95% (*v*/*v*) cold ethanol and maintained at 4 °C for 24 h. After spinning at 8000 r/min for 40 min at 4 °C, the EPS precipitation was obtained and resuspended in 100 mL of deionized water (DI water). Next, an equal volume of 10% TCA (*v*/*v*) was added to the EPS solution and kept at 4 °C for 24 h. After spinning at 8000 r/min for 40 min at 4 °C, the protein precipitate was removed, and then the ethanol participation and centrifugation at the same condition were repeated again. The EPS was dialyzed (membrane cut-off 14,000) at 4 °C for 48 h (water changed after 8 h) [21]. Finally, EPS715 was collected and lyophilized for further study.

### 2.3. Spectral Analysis

The lyophilized EPS715 was dissolved in DI water (1 mg/L), and scanning was performed at 200–500 nm using a UV–Vis spectrophotometer [22].

FTIR was mainly employed to evaluate the type of glycosidic bonds in the EPS and observe functional groups, according to Xu et al. [23]. Precisely, freeze-dried EPS715 (2 mg) was ground using 200 mg dry KBr powder and then scanned within the 4000 to 400 cm^−1^ range (Nicolet 6700, Thermo Scientific, Waltham, MA, USA) with 4 cm^−1^ resolutions, a step width of 2 cm^−1^, and 32 scans.

### 2.4. Detection of the Molecular Weight (Mw)

Gel permeation chromatography (GPC) was employed to observe the Mw of EPS715 using a PL aquagel-OH MIXED column (8 μm). The same previous procedure was followed with slight modification [24]. Before injection, the sample (5 mg/mL) was filtered via a 0.22 μm filter membrane. There was a 10 μL injection volume. The column was eluted at 1 mL/min (flow rate) with 0.2 mol/L NaNO_3_ and 0.01 mol/L NaH_2_PO_4_ at 30 °C.

### 2.5. Monosaccharide Composition Analysis

The composition of EPS715 was detected via high-performance liquid chromatography (HPLC) [13]. First, the EPS715 sample (5 mg) was added into a 5 mL sealed test tube with 1 mL 2 mol/L trifluoroacetic acid (TFA), which was hydrolyzed at 121 °C for 2 h and then kept at room temperature (RT). The hydrolyzed sample was dissolved with DI water to 50 mL, filtered by a 0.45 μm membrane, and analyzed by HPLC using a Shimazu LC-20AD (Tokyo, Japan) with a C18 column (4.6 × 200 mm, 5 um) (Elite, Dalian, China). Next, 20 µL of the aqueous phase (detected at 250 nm) was injected, and the temperature of the column was 30 °C. The split ratio of mobile phase A (0.05 mol/L KH_2_PO_4_, pH was set at 6.7 by 1 mol/L NaOH) and mobile phase B (acetonitrile) was 83:17, and the flow rate was 1 mL/min. Mannose, rhamnose, ribose, galacturonic acid, glucuronic acid, glucose, N-acetyl-glucosamine, galactose, N-acetyl- xylose, galactose, arabinose, and fucose were selected as standards and tested under the same conditions.

### 2.6. Congo Red Experiment

The Congo red experiment is usually performed to identify the triple-helix structure of EPSs. The same procedure was followed as outlined by Guo et al. [25] with slight changes. Briefly, Congo red (1 mg/mL) was mixed with EPS (1 mg/mL) solutions in an equal volume. The NaOH solution (1 mol/L) was added to produce a series of solutions with different NaOH (0.1 to 0.5 mol/L) concentrations and kept at RT in the dark for 5 min. The maximum absorption wavelength (λ_max_) of the mixture was scanned at 400 to 700 nm. Congo red and water without EPS were used as the control. Next, we plotted the λ_max_ versus NaOH concentration.

### 2.7. Scanning Electronic Microscope (SEM) Analysis

The surface structure of the EPS was analyzed via SEM (Zeiss sigma 300, Oberkochen, Germany). The freeze-dried EPS was adhered on the SEM stubs with double-sided adhesive and coated with a layer of gold (approximately 10 nm). The surface structure of EPS was performed via SEM using a 10 kV accelerating voltage at different magnifications (400×, 1000×, 2000×) [26].

### 2.8. X-Ray Diffraction (XRD) Analysis

Lyophilized EPS715 was placed in the sample section of the device. The XRD profiles were obtained using a Bruker D8 Advance XRD (Bruker, Saarbrucken, Germany), operating at a voltage of 40 kV and a current of 40 mA. The diffraction levels were measured on EPS715 across a range of 5 to 100° with a step size of 0.02° in the 2θ range [27].

### 2.9. Particle Size and Zeta Potential

The lyophilized EPS715 was reconstituted to a 1.0 mg/mL concentration using distilled water [28]. The zeta potential, average particle size, size distribution, and polydispersity index (PDI) were assessed by a Brookhaven NanoBrook 90Plus Zeta instrument (Brookhaven Instruments Corporation, New York, NY, USA). All of these parameters were measured at RT.

### 2.10. Differential Scanning Calorimeter (DSC) Analysis

The thermal parameters of EPS were detected via differential scanning calorimetry (DSC 8000, PerkinElmer, Shelton, CT, USA). Approximately10 mg of EPS was weighed into an aluminum pan and pressed down to seal the pan. The samples were evaluated at a heating rate of 10 °C/min, in a nitrogen atmosphere, within the 20 to 500 °C temperature range [29].

### 2.11. In Vitro Antioxidant Activities

#### 2.11.1. ABTS^+^ Scavenging Assay

The radical scavenging activity of EPS was evaluated using the 2,2′-azinobis (3-ethylbenzothiazoline-6-sulfonate) (ABTS) method as outlined by Kang et al. [30], with some modifications. Precisely, radical cations were prepared by mixing ABTS (7 mM) with potassium persulfate (2.45 mM) (1:1, *v*/*v*), and this mixture was kept in the dark (RT) for 24 h. The absorbance of the ABTS^+^ solution was set at 0.7 ± 0.02 at 734 nm. Next, 500 μL EPS (0.625, 1.25, 2.5, 5 mg/mL) and 1000 μL ABTS^+^ solution were mixed and kept for 10 min incubation at RT in the dark. The mixture absorbance (OD) was noted at 734 nm. The clearance rate was calculated via Equation (1):

(1) Scavenging effect (%)=(Ab−As)/Ab × 100

where the OD of the blank and sample group was denoted by A_b_, and A_s_, respectively.

#### 2.11.2. DPPH Scavenging Assay

Briefly, DPPH-ethanol (0.2 mmol/L) solution was added to different concentrations of EPS with an equal volume, kept at 25 °C in the dark for 30 min incubation, and then the mixture was spun. After that, the OD of the supernatant was determined at 517 nm. The blank samples consisted of DI water. The scavenged DPPH radical was calculated in percentage using Equation (2) [31]:

(2) Scavenging effect (%)=(1−As/Ab) × 100

where A_s_ and A_b_ represent the OD of the sample and blank groups, respectively.

#### 2.11.3. Hydroxyl Radical (·OH) Quenching Assay

The OH quenching ability was determined by using the same method as previously mentioned [23]. Briefly, 1 mL PBS (pH 7.4), 0.5 mL 1,10-phenanthroline (0.25 mmol/L), 0.5 mL ferrous sulfate heptahydrate (2.5 mmol/L), and 0.5 mL of the sample solution were mixed (equal volume of PBS solution was added into the control group). After that, H_2_O_2_ (0.5 mL, 20 mmol/L) was added. Then, the mixture was kept for 60 min at 37 °C, and the OD was noted at 536 nm. The scavenging potential of OH radicals was calculated as Equation (3).

(3) Hydroxyl radical scavenging effect (%)=[(As−Ab)/(Ac−Ab)]× 100

The OD of the sample, blank, and control group was denoted by A_s_, A_c_, and A_b_, respectively.

#### 2.11.4. Reducing Power Assay

The same procedure described by Adebayo-Tayo et al. [32] was followed to analyze the reducing power assay with minor modifications. Precisely, 500 μL of potassium ferricyanide (1%, *w*/*v*) and 500 μL of phosphate buffer (2 mM, pH 6.6) were mixed with 200 μL of EPS (0.625, 1.25, 2.5, 4, 5 mg/mL). The mixture was maintained at 50 °C in a water bath for 20 min. The reaction mixture was spun at 3000 × g for 10 min after adding 500 μL of 10% trichloroacetic acid. The supernatant was inoculated into 2.5 mL of distilled water and 0.1 mL of ferric chloride (1%, *w*/*v*) and kept for 10 min incubation. The readings were obtained at 700 nm, with different ascorbic acid concentrations as a positive control.

Reducing power assay = A_s_ − A_b_ (4)

where the OD of the sample and blank group was denoted by A_s_ and A_b_, respectively.

### 2.12. In Vitro Antibiofilm Activities

#### 2.12.1. Biofilm Inhibition Assay

Approximately 180 μL of LB broth was added to 20 μL of sterile EPS715 at different concentrations (0.375, 0.625, 1.25, 2.5, and 5 mg/mL). Five pathogenic bacteria including *S. aureus* (preserved by our laboratory), *B. cereus* (isolated from the spoilage shrimp paste), *S. saprophyticus* (isolated from the spoilage shrimp paste), *Acinetobacter* spp. (isolated from the spoilage fish), and *H. alvei* (isolated from the spoilage salmon) were selected as test strains. The overnight cultures of the pathogens were inoculated (2%, *v*/*v*) into each well and incubated statically at 37 °C for 24 h. Specifically, the plates containing *Acinetobacter* spp. and *H. alvei* were kept at 28 °C for 24 h incubation. Next, a crystal violet assay was performed to evaluate the antibiofilm activities. Briefly, the cultures were removed, and the wells were rinsed with PBS (0.2 mL, pH 7.2) and dried with air. The biofilm was stained with 0.1% crystal violet (200 μL) for 30 min at RT, and then washed with PBS 2–3 times. The crystal violet was dissolved with 200 μL of 95% ethanol for 30 min, and the OD of each well was noted at 595 nm using a microplate reader. The inhibition rate was estimated via Equation (5) [33].

Inhibition rate (%) = (OD_control_ − OD_sample_)/OD_control_ × 100 (5)

#### 2.12.2. The Impact of EPS on Preformed Bacterial Biofilm

The bacterial biofilms were formed in the same conditions as described above. The wells were washed with 200 μL of PBS/well to remove nonadherent cells. Next, 180 μL of the LB medium and 20 μL of different concentrations of EPS715 were transferred into each well. The wells without EPS715 were used as the control. The plates were then cultivated at 37 °C for 24 h. Next, the media were removed, and the wells were washed thrice with sterile PBS. As previously reported, the biofilm biomass was evaluated using a crystal violet assay and calculated by Equation (6).

Dispersion rate (%) = (OD_control_ − OD_sample_)/OD_control_ × 100 (6)

### 2.13. The Analysis of the Antibiofilm Mechanism of EPS715 on H. alvei

#### 2.13.1. Effects of EPS715 on the Growth of *H. alvei*

*H. alvei* was inoculated (2%, *v*/*v*) into LB broth including 1 mL sterile EPS715 solution (5 mg/mL), making the final concentration of EPS715 in the tube 500 μg/mL. Wells without EPS715 was considered the negative control, the OD _600 nm_ of the bacterial suspension within 24 h was measured every 2 h, and the growth curve of the bacteria was made according to the time and the value of the OD_600 nm_.

#### 2.13.2. Effects of EPS715 on the Auto-Aggregation and Hydrophilic Ability of *H. alvei*

To examine the possible mechanism of the EPS antibiofilm, an auto-aggregation test was conducted between EPS715 and *H. alvei* [13]. Briefly, *H. alvei* was cultured overnight in LB broth and spun at 8000× *g* for 10 min. After removing the supernatant, cells were rinsed with PBS, spun again, and resuspended in PBS. The OD_600_ was adjusted to 1.0 ± 0.2. The culture suspension (1 mL) was mixed with EPS715 (final concentration 500 μg/mL) as the treated group and the suspension without EPS715 as the blank control. Both suspensions were vigorously shaken with a vortex for 2 min, and the absorbance was promptly quantified at 600 nm (t0). The suspension’s absorbance OD_600_ nm was determined at different time points (1, 2, 3, and 24 h) and indicated as t_1_, t_2_, t_3_, and t_24_, respectively. The EPS effect on the hydrophobicity of *H. alvei* was observed by their adhesion to liquid hydrocarbons such as xylene. Briefly, EPS715 (1 mg/mL) was mixed with *H. alvei* suspension (2.4 mL) and vortex for 2 min, then measured at OD_600 nm_ (A_0_). Next, 0.5 mL of xylene was added and vortexed for 2 min. The mixture was equilibrated at ambient temperature for 30 min. Finally, 1 mL of each suspension was extracted from the lower aqueous phase and analyzed at OD_600 nm_. The hydrophobicity was calculated by Equation (7).

Hydrophobicity = [(A_0_ − A_t_)/A_0_] × 100 (7)

where A_0_ and A_t_ are the absorbance of the suspension measured before and after mixing, respectively.

#### 2.13.3. Effects of EPS715 on the Swarming and Swimming Ability of *H. alvei*

The overnight cultured strains (OD_595 nm_ = 0.5) were directly inoculated at the center of swarming agar (1% tryptone, 0.5% NaCl, 0.5% glucose, and 0.6% agar) and swimming agar (0.5% NaCl, 1% tryptone, and 0.3% agar) containing 62.5 μg/mL or 500 μg/mL EPS715 or without EPS715. The culture was maintained at 28 °C for 24 h. After incubation, the zones of migration originating from the inoculation site were used to assess their swarming and swimming capabilities.

### 2.14. Statistical Analysis

Data were statistically examined via SPSS 26.0 software and evaluated by one-way ANOVA and Duncan test. All experiments were repeated independently at least in triplicate, and the results were illustrated as mean ± SD. A significance level was set when the value of *p* < 0.05.

## 3. Results and Discussion

### 3.1. Characterization of EPS715

#### 3.1.1. Spectra Analysis

**Figure 1:**
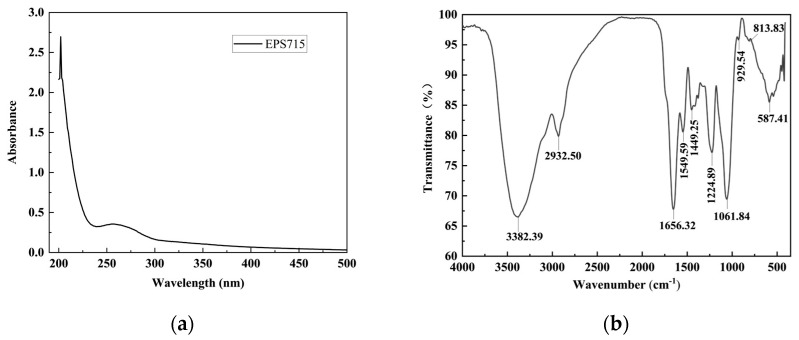
Spectral analysis of EPS715. (**a**) UV–Vis spectra; (**b**) FTIR spectra.

UV–Vis spectroscopy was used to identify the existence of nucleic acids (NAs) and proteins in the EPS. The UV–Visible spectrum of EPS is depicted in Figure 1a. The λ_max_ wavelength of EPS715 was around 200 nm, which is the typical absorption peak of polysaccharides [24]. The absence of absorption peaks (260 and 280 nm) indicates no NAs and protein in EPS715 [34].

The Fourier transform infrared (FTIR) spectra of EPS715 are depicted in Figure 1b. The peak identified within the 3000–3500 cm^−1^ wavenumber spectrum was ascribed to the presence of O-H bonding. The prominent peak at 3382.39 cm^−1^ can be attributed to the vibrational stretching of the O-H bond. The stretching vibration peak observed at 2932.50 cm^−1^ was identified as the result of the stretching vibration of the C-H bond [35]. The identification of the previously mentioned unique absorption peaks indicates that the sample is composed of carbohydrates. The absence of a visible absorption peak in the 1700–1775 cm^−1^ range indicates that a carboxyl group was not present in the sample. Thus, EPS715 can be classified as a neutral polysaccharide. The absorption bands observed at 1650 to 1540 cm^−1^ were related to the stretching vibrations of the enol and amide II groups [36]. These vibrations are depicted in the spectra at 1549.59 and 1656.32 cm^−1^. Furthermore, the distinct peak observed at 1656.32 cm^−1^ was identified as being caused by the presence of bound water and can be related to the existence of C-O bonds. The spectral range of 1200–950 cm^−1^ is commonly called the “fingerprint region” or “sugar region” of polysaccharides. This term describes the detailed structure of polymers like polysaccharides. The absorption peaks seen in the 1000–1200 cm^−1^ range were specifically related to the C-O-C and C-O-H bonds of pyranose [37]. The existence of a C-O-C bond led to the detection of an absorption peak at 1061.84 cm^−1^. The lack of a peak at 890 cm^−1^ indicates the absence of a β-configuration in the EPS sample [38]. Furthermore, the absorption peak at 810 cm^−1^ confirmed the presence of Man [23], which is in line with the results of the monosaccharide composition.

#### 3.1.2. Molecular Weight (Mw) and Monosaccharide Composition Analysis

**Figure 2:**
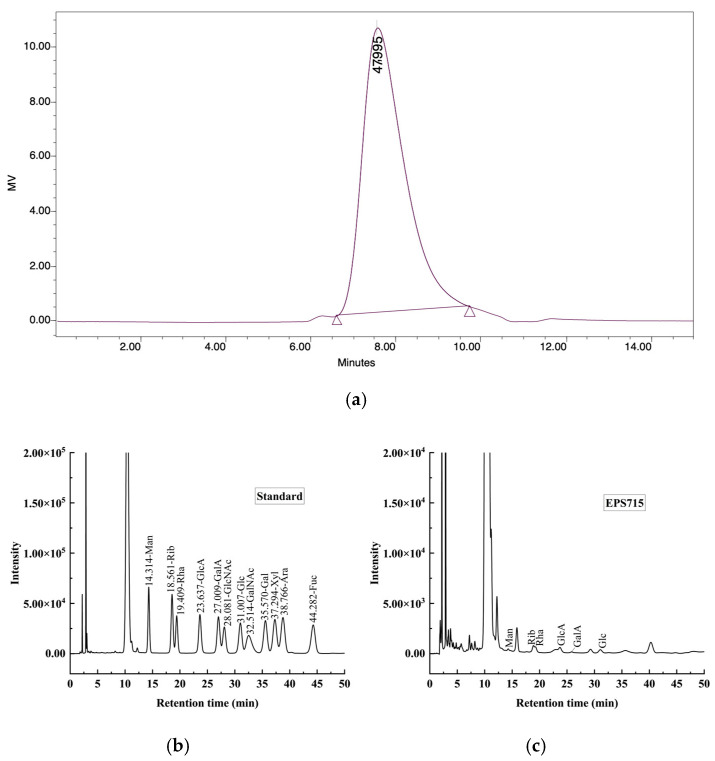
The Mw and monosaccharide composition of EPS715. (**a**) GPC chromatogram of EPS715. The peak width was marked in the spectragram. (**b**) HPLC spectrum of the standard. (**c**) HPLC spectrum of the EPS hydrolates.

The GPC analysis showed that EPS715 demonstrated a Mw of 4.79 × 10^4^ Da (Figure 2a). It was reported that the EPS from LAB possessed Mw in the range of 4.0 × 10^4^ − 6.0 × 10^6^ Da [39]. The Mw of EPS secreted by *L. plantarum* C70 was 3.8 × 10^5^ Da [40], and the glucan-DM5 from *L. plantarum* DM5 had a higher Mw of 1.11 × 10^6^ Da [41]. Previous studies found that low Mw polysaccharides usually had good antioxidant activity [42].

As shown in Figure 2b,c the HPLC results revealed that EPS715 contained glucose, mannose, and rhamnose, with a molar ratio of 2.73:0.56:3.32, which indicated that EPS715 is a Hep. The EPS from *L. plantarum*, mainly composed of glucose and galactose, has also commonly been reported [43,44,45]. Moreover, rhamnose and mannose were also present, similar to the current results. Glucose and rhamnose were the predominant monosaccharides in EPS715. Rhamnose may be beneficial for the antibacterial function of polysaccharides; Han et al. [46] suggested that BPP-3 with high contents of rhamnose and uronic acid enhanced the antibacterial effects against *E. coli*, *Bacillus subtilis*, *Staphylococcus aureus*, and *Pseudomonas aeruginosa* compared with BPP-1 and BPP-2. Mannose may regulate immune activity by activating macrophages. The content of uronic acid may be directly proportional to antioxidant activity [47].

#### 3.1.3. Congo Red Test

**Figure 3:**
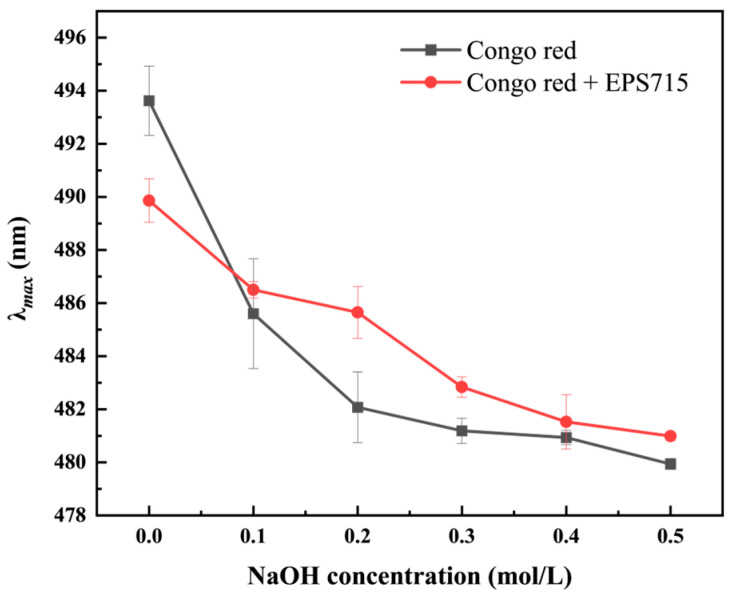
Results of the Congo red experiment of EPS715.

Congo red can form complexes with polysaccharides in weakly alkaline solutions, causing a red shift (toward the long-wave direction) of the absorption maximum (λ_max_) compared to Congo red in the NaOH solution [48]. The results showed that EPS715 had no triple-helix structure (Figure 3), which is similar to that found in a previous study [24]. The presence of the triple-helix structure may have an effect on anticancer activity [49]. Generally, low molecular weight polysaccharides do not have a triple-helix structure, but rather a single stranded helix structure [50]. In addition, HePs generally have no triple-helix structure [51]. The results of the Congo red test were consistent with the results of Mw and the monosaccharide composition of EPS715.

#### 3.1.4. Microstructural and Thermal Characteristic Analysis

**Figure 4:**
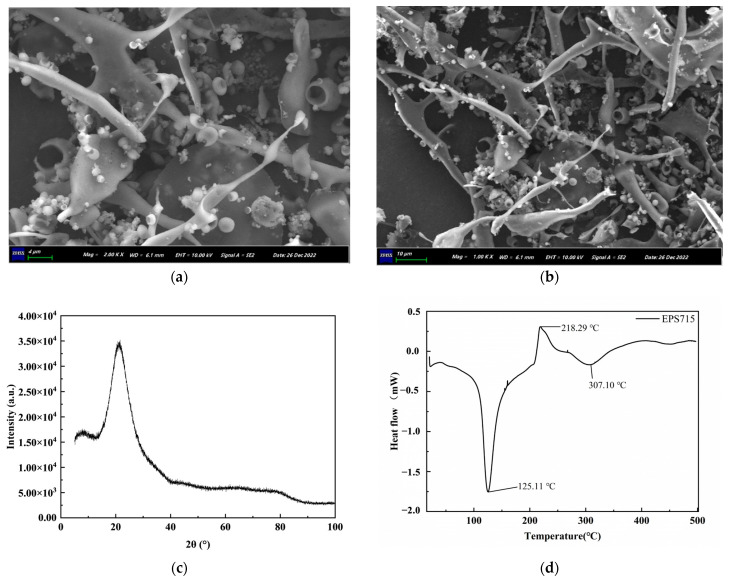
Microstructural and thermal characteristic analysis of EPS715. (**a**) SEM image of EPS715 at 1000×. (**b**) SEM image of EPS715 at 2000×. (**c**) XRD pattern. (**d**) DSC of EPS715.

The microstructure of EPS715 is shown in Figure 4a,b. The EPS715 was reticulated and had multiple branches and holes. The pores in the surface structure will loosen the surface structure of polysaccharides, increase their surface area, and ultimately increase their solubility. For example, the EPS from *L. plantarum* YW32 may exhibit better rheological properties and good water solubility due to its compact surface structure and multiple pores [45]. Compared to oil-soluble substances, water-soluble substances are usually more widely used, and this solubility is conducive to the widespread application of EPSs in food. The amorphous or crystalline nature of EPS715 was investigated using X-ray diffraction (XRD). A vast diffraction peak at about 2θ 21.38°, as depicted in Figure 4c, suggests the amorphous and non-crystalline nature of EPS715 [27,34]. This observation agrees with the scanning electron microscope (SEM) image.

The particle size distribution of EPS715 ranged from 30 to 1000 nm with an average particle size of 853.66 ± 23.54 nm, and its polydispersity coefficient (PDI) was found to be 0.328. The average zeta potential of EPS715 was −11.49 ± 1.60 mV. The zeta potential of EPS715 was found to be negative, indicating the presence of a negative charge and the ability to donate electrons. This could be linked to uronic acid in polysaccharides [37]. In addition, the absolute value of zeta potential is directly proportional to the stability of the solution [28]. The thermal characteristics of EPS715 were assessed by DSC analysis. The DSC thermogram of EPS715 exhibited distinct endothermic peaks at 125.11 °C and 307.10 °C (Figure 4d), representing its melting point (Tm) at 125.11 °C. It was shown that the Tm of *L. fermentum* A51-EPS-1 was 84.2 °C [52]. A higher Tm is beneficial for the application of polysaccharides as food additives in baked food.

### 3.2. In Vitro Antioxidant Activities of EPS

**Figure 5:**
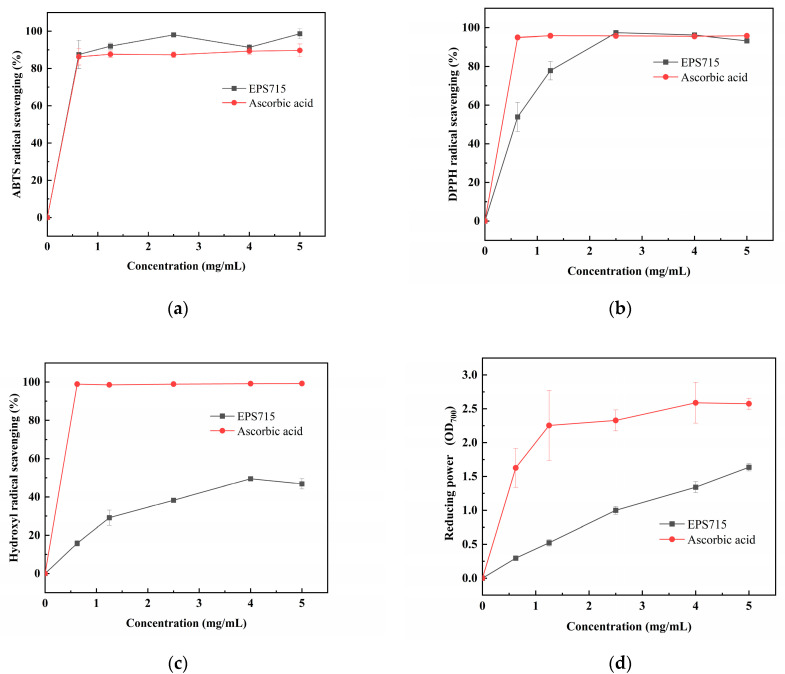
In vitro antioxidant activities of EPS715. (**a**) ABTS scavenging ability. (**b**) DPPH scavenging ability. (**c**) Hydroxyl radical scavenging. (**d**) Reducing power.

Figure 5 demonstrates the ability of EPS715 to scavenge various free radicals. In general, EPS715 exhibited remarkable antioxidant properties. The scavenging rates of EPS715 toward ABTS ranged from 87.48 ± 7.63% to 98.64 ± 2.70%, and a concentration of 5 mg/mL of EPS715 exhibited the highest scavenge ability (Figure 5a). EPS715 also exhibited a commendable scavenging efficacy against DPPH and hydroxyl radicals. The investigation showed that EPS715 at a concentration of 2.5 mg/mL exhibited the most significant DPPH scavenging effect, with a 97.37 ± 0.79% scavenging rate (Figure 5b). EPS715 exhibited a lower efficacy in scavenging hydroxyl radicals compared to DPPH and ABTS. However, the IC50 of EPS715 was 4 and 5 mg/mL (Figure 5c). As depicted in Figure 5d, EPS715 also displayed notable reducing activity and concentration dependence. The reducing capacity of EPS715 at 5 mg/mL was observed to be 1.64 ± 0.05 absorbance at 700 nm. With respect to the ascorbic acid standards, EPS715 was equivalent to 0.629 mg/mL of ascorbic acid. The antioxidant activity of the EPS is significantly affected by its monosaccharide composition, proportion, functional groups, and net charge [53,54]. The results show that EPS715 had good antioxidant activity and can be used as a potential antioxidant of natural origin, which provides a theoretical basis for future application in the field of food and pharmaceutical engineering.

### 3.3. In Vitro Antibiofilm Activities of EPS715

The impact of EPS715 on the formation of biofilms by five pathogens is shown in Table 1 and Table 2. EPS715 exhibited a substantial inhibitory impact on the biofilm of *Bacillus cereus* and *H. alvei*, achieving the greatest inhibition rates of 73.29% and 78.17%, respectively. However, the concentration of EPS at 37.5 μg/mL was insufficient to prevent the formation of biofilm by *S. aureus*. The greater antibiofilm activity was detected against *S. aureus* with inhibition percentages of 60.08 ± 12.98%, corresponding to EPS715 of 125 μg/mL. The inhibition rate of EPS715 on *Staphylococcus saprophyticus* was 8.05%–54.13%. The inhibitory effect against *Acinetobacter* spp. biofilm formation was relatively subdued for EPS715. In addition, the dispersion effect of EPS715 on biofilm was higher than the inhibitory effect. As shown in Table 2, the highest dispersion effect of EPS715 on the biofilm of five bacterial strains ranged from 36.88% to 84.01%. Among all of the strains, the dispersion effect on *B. cereus* was the highest (84.01%), followed by *S. aureus* (83.47%), *H. alvei* (78.92%), and *S. saprophyticus* (62.35%), while EPS715 only exhibited activity with 36.88% dispersion of the *Acinetobacter* spp. biofilm. Overall, the inhibitory and dispersive effects against Acinetobacter spp. biofilm were relatively subdued.

Other researchers have also reported on the antibiofilm effect of the EPS produced by *L. plantarum*, and the inhibition activities relate to the origin of the strain, the type of EPS (such as monosaccharide composition and the structure), and the species of pathogen. It was found that EPS produced by milk-originated *L. plantarum* WLPL04, mainly containing xylose, glucose, and galactose, had an antibiofilm effect against *Pseudomonas aeruginosa* CMCC10104, *E. coli* O157:H7, *Salmonella Typhimurium* ATCC13311, and *S. aureus* CMCC26003, and the inhibition rate was higher for *P. aeruginosa* than *E. coli* [55]. EPS-7, from fermented rice gruel-originated *L. plantarum* PRK7, had antibiofilm abilities against *Micrococcus luteus*, *S. aureus*, *P. aeruginosa*, *Streptococcus pneumoniae*, *Klebsiella pneumoniae*, and *E. coli*, moreover, its antibiofilm effect was stronger than another EPS produced by another *L. plantarum*, which was also isolated from fermented rice gruel, due to the fibrous morphology of EPS-7 [56]. For the EPS of *L. plantarum* 12, which was isolated from kimchi, it could inhibit the biofilm formation of *Shigella flexneri*; in addition, its acidic polysaccharide component L-EPS 2-1 had a higher antibiofilm effect than the neutral component [57].

### 3.4. Potential Antibiofilm Mechanism of EPS715 on H. alvei

**Figure 6:**
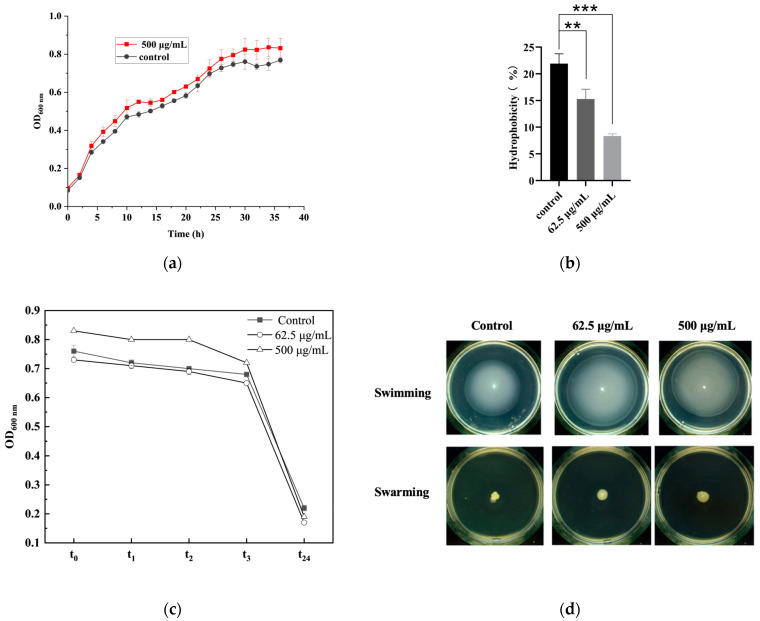
The potential mechanism of EPS715 against *H. alvei*. (**a**) The effect of EPS715 on the growth of *H. alvei*. (**b**) The effects of EPS715 on the hydrophobicity of *H. alvei*. (**c**) The effects of EPS715 on the self-aggregation of *H. alvei*. (**d**) The effects of EPS715 on the swimming and swarming ability of *H. alvei.* ** (*p* < 0.01), *** (*p* < 0.001).

EPS715 did not affect the growth rate of the test strain (Figure 6a). This result indicates that the inhibition of EPS715 against the *H. alvei* biofilm was not due to an antibacterial effect, which is in accordance with the characteristics of some EPSs such as the EPS from *L. plantarum* 12, which did not have an antibacterial effect toward *S. flexneri* [57]. However, there other reports have proven that some *L. plantarum* EPSs have strong antibacterial effects such as L-EPS from *L. plantarum* R315, which could inhibit the growth of *Cronobacter sakazakii*, *E. coli*, *Listeria monocytogenes*, *S. aureus*, *Candida albicans*, *Bacillus cereus*, *S. typhimurium*, and *Shigella sonnei* [58] as well as the EPS-Lp produced by cheese originated *L. plantarum*, which has an antibacterial effect against *E. coli* [59].

The formation of the biofilm includes several steps: initial attachment of the bacteria, bacterial aggregation, maturation, and dispersion of the biofilm [60]. The aggregation ability of bacteria and the hydrophobicity of cell surfaces could interfere with the adhesion process. These two abilities were considered important factors inhibiting biofilm formation [13,61]. As shown in Figure 6b,c, for *H. alvei*, the binding affinity toward xylene was significantly reduced when incubated with EPSs of different concentrations (62.5 μg/mL and 500 μg/mL). In addition, high concentrations of EPS715 may also have had an inhibitory effect on the auto-aggregation of *H. alvei* (Figure 6c). EPS715 (500 μg/mL) may inhibit the auto-aggregation of *H. alvei* when co-incubated in the first 3 h. However, 62.5 μg/mL EPS715 had almost no inhibition effect on the auto-aggregation of *H. alvei*. The EPS from *L. plantarum* EIR/IF-1 could also inhibit the binding ability of the pathogens to hydrocarbons and aggregation of the pathogens by reducing bacterial hydrophobicity, which acts as the antibiofilm mode of the surfactant [13].

In addition, bacteria’s swimming ability was beneficial for the formation of biofilms, which was attributed to their ability to form cell clusters and aggregate on the surface of the matrix for movement. It was found that EPS715 could improve the strain’s swimming ability but had no effect on its swarming ability (Figure 6d). The increase in swimming ability would delay the formation of the biofilm and accelerate dispersion of the biofilm.

The extracellular polysaccharides produced by one microorganism can act as exogenous substances to another, triggering a stress response in the latter that prevents internalization. This interaction leads to changes in the expression levels of genes and proteins involved in various biological processes within the cells. Consequently, the role of extracellular polysaccharides in regulating key genes and signaling pathways related to bacterial biofilm formation has garnered increasing attention in recent years. For instance, the extracellular polysaccharide secreted by *L. helveticus* MB2-1, derived from yogurt and containing fucose, has been shown to significantly downregulate the expression of the *agrA* gene, which is closely linked to biofilm formation as well as the important regulatory factor *sarA* and the sigma factor sigB in methicillin-resistant *S. aureus* [62]. Furthermore, the extracellular polysaccharide EPS273, with a molecular weight of 190 kDa and comprising glucosamine, rhamnose, glucose, and mannose, secreted by the marine bacterium Pseudomonas stutzeri 273, downregulates genes associated with the PhoP-PhoQ two-component signaling system, *lasI*/*lasR,* and *rhlI*/*rhlR* in *P. aeruginosa*. Notably, the absence of phoP and phoQ genes can significantly affect the biofilm formation and motility of Pseudomonas aeruginosa [63,64]. Although EPS715 could impact the swimming ability of *H. alvei*, which might be controlled by the cell signaling regulatory system, the exact effect of EPS715 on the cell signaling regulatory system needs to be further investigated.

## 4. Conclusions

The EPS was extracted from the pickled vegetable originated *L. plantarum* PC715. The structural characteristics of EPS715 were analyzed with FTIR, GPC, HPLC, SEM, XRD, and DSC. The ABTS, DPPH, hydroxyl radical scavenging activities, and reducing power assay were exhibited by EPS715. EPS715 could inhibit biofilm formation and disperse the biofilms of *S. aureus*, *B. cereus*, *S. saprophyticus*, *Acinetobacter* spp., and *H. alvei*. Therefore, EPS produced from *L. plantarum* PC715 has the potential to be used as an antibiofilm agent. This can be related to the inhibitory effects of EPS on bacterial auto-aggregation and bacterial binding to hydrocarbons. In addition to cell surface adhesion, further experiments are needed to explore the molecular inhibition mechanisms, especially the relationship with quorum-sensing genes. Our research also proved that the antibiofilm of the EPS has a relationship with the origin of the EPS-producing strain, the structure, and the pathogen species. In future studies, the relationship between antibiofilm function and the structure of the *L. plantarum* EPS also needs to be investigated in depth.

## Figures and Tables

**Table 1:** Biofilm inhibition rate of EPS715 against different pathogens.

EPS Concentration (μg/mL)	Inhibition Rate (%)				
	*S. aureus*	*B. cereus*	*S. saprophyticus*	*Acinetobacter* spp.	*H. alvei*
37.5	−6.78 ± 21.68% ^b^	66.51 ± 7.11% ^ab^	39.16 ± 13.68% ^a^	18.69 ± 4.14% ^a^	58.38 ± 2.06% ^b^
62.5	32.49 ± 11.82% ^a^	73.29 ± 3.71% ^a^	34.38 ± 10.33% ^a^	29.20 ± 10.96% ^a^	78.17 ± 1.65% ^a^
125	60.08 ± 12.98% ^a^	41.19 ± 10.99% ^bc^	8.05 ± 16.51% ^b^	10.31 ± 14.50% ^a^	44.01 ± 0.44% ^c^
250	56.92 ± 7.42% ^a^	38.93 ± 3.76% ^bc^	54.13 ± 0.93% ^a^	23.33 ± 20.90% ^a^	30.31 ± 1.19% ^d^
500	53.28 ± 15.62% ^a^	26.74 ± 26.14% ^c^	46.82 ± 0.90% ^a^	18.47 ± 7.49% ^a^	32.06 ± 5.40% ^d^

Different lowercase letters indicate significant differences between concentrations for different pathogens (*p* < 0.05).

**Table 2:** The impact of EPS715 on preformed pathogenic biofilms.

EPS Concentration (μg/mL)	Dispersion Rate (%)				
	*S. aureus*	*B. cereus*	*S. saprophyticus*	*Acinetobacter* spp.	*H. alvei*
37.5	75.51 ± 1.95% ^b^	73.24 ± 4.46% ^a^	62.35 ± 2.29% ^a^	4.10 ± 11.48% ^c^	35.66 ± 11.02% ^c^
62.5	83.47 ± 0.84% ^a^	82.18 ± 4.55% ^a^	35.14 ± 10.65% ^bc^	19.30 ± 7.60% ^bc^	36.38 ± 4.86% ^c^
125	50.03 ± 3.34% ^c^	84.01 ± 3.04% ^a^	24.97 ± 8.99% ^c^	36.88 ± 2.33% ^a^	65.86 ± 1.23% ^b^
250	72.80 ± 2.07% ^b^	81.39 ± 2.62% ^a^	51.13 ± 2.25% ^ab^	21.22 ± 5.65% ^ab^	60.75 ± 4.33% ^b^
500	75.20 ± 2.66% ^b^	64.80 ± 16.21% ^a^	−26.46 ± 8.69% ^d^	27.61 ± 4.09% ^ab^	78.92 ± 2.05% ^a^

Different lowercase letters indicate significant differences between concentrations for different pathogens (*p* < 0.05).

## Data Availability

The data will be available by the corresponding authors on request.

## Supplementary Materials

The following supporting information can be downloaded at: https://www.mdpi.com/article/10.3390/microorganisms12112229/s1, Figure S1: The production of the EPS on the MRS plate.
