# Supplementary figures and images for: Characterization of Exopolysaccharides from Lactiplantibacillus plantarum PC715 and Their Antibiofilm Activity Against Hafnia alvei
Source: Microorganisms. 2024 Nov 3;12(11):2229. doi: 10.3390/microorganisms12112229 (PMC11596824; doi:10.3390/microorganisms12112229)

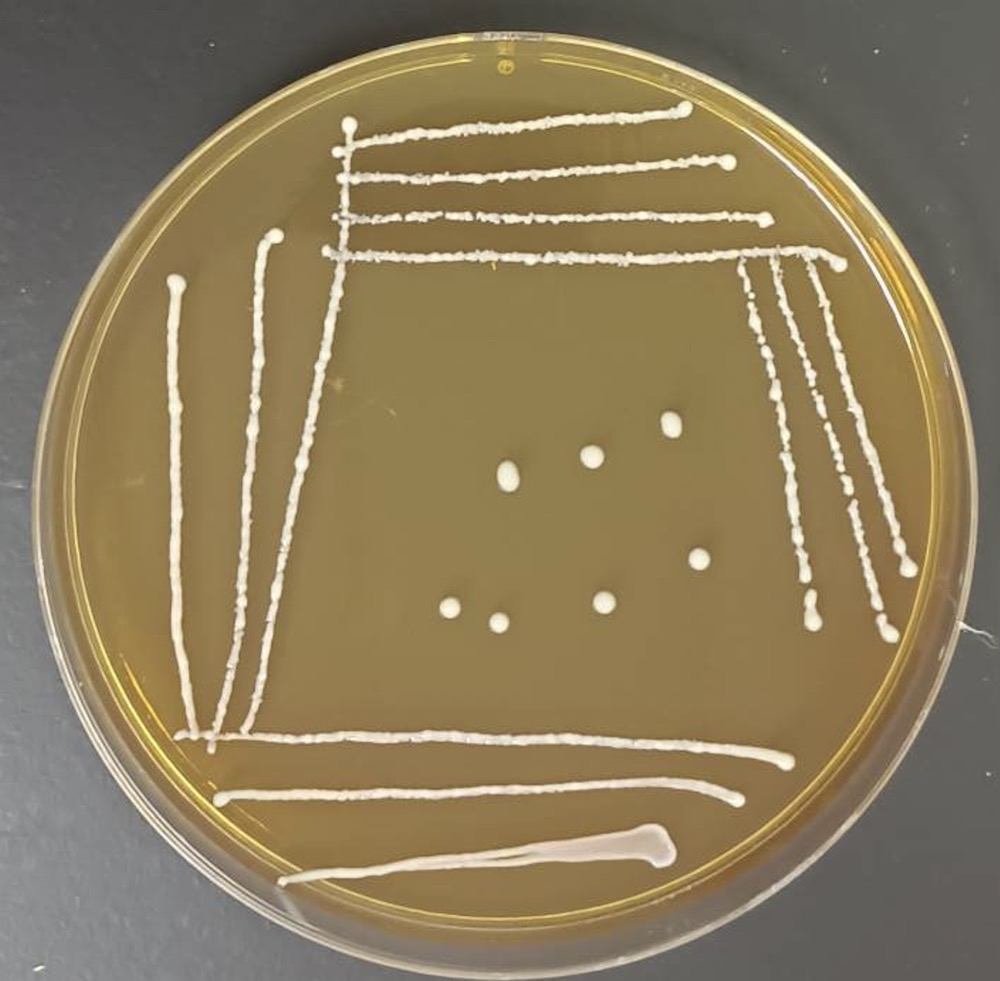

Supplement: Supplementary file 1 [file microorganisms-12-02229-s001.zip › microorganisms-3240026-supplementary.jpg]
